# A Sutureless Approach to Nerve Repair: Results From a Clinical Study in Digital Nerves

**DOI:** 10.1016/j.jhsg.2026.100956

**Published:** 2026-02-27

**Authors:** Randy Bindra, Michael Wagels, Marie-Elena Brett, Dominic Power

**Affiliations:** ∗ Department of Orthopaedic Surgery, Griffith University School of Medicine and Dentistry, Southport Gold Coast, Queensland, Australia; † Department of Plastic and Reconstructive Surgery, Princess Alexandria Hospital, Woolloongabba, Queensland, Australia; ‡ TISSIUM, Cambridge, MA; § The Peripheral Nerve Injury Service, Queen Elizabeth Hospital, Birmingham, England, UK

**Keywords:** COAPTIUM Connect, Digital nerve injury, Peripheral nerve repair, Polymer-assisted nerve repair, Sutureless coaptation

## Abstract

**Purpose:**

Traditional microsurgical suture neurorrhaphy presents challenges such as fascicular trauma, scar formation, and variability in repair quality. This clinical study evaluates the safety and efficacy of a bioresorbable poly(glycerol sebacate) acrylate (PGSA) polymer-assisted device in digital nerve repair without sutures.

**Methods:**

A single-arm clinical trial enrolled 12 patients with digital nerve injuries at two centers. The repair involved securing severed nerve ends within a 3-dimensional printed PGSA coaptation chamber using a light-activated PGSA polymer. Patients were prospectively followed for 12 months with assessments including pain (visual analog scale pain score), sensory recovery (Semmes-Weinstein Monofilament and two-point discrimination), patient-reported outcomes (Impact of Hand Nerve Disorders Questionnaire v2.0), and high-resolution ultrasound imaging.

**Results:**

At 12 months, 10 patients who completed the study had a Semmes-Weinstein Monofilament score of 0.4 (“diminished light touch”) or better, with 100% achieving “Good” (S3+) or “Excellent” (S4) two-point discrimination scores. No patient reported pain at 12 months, and no device-related complications were recorded. Ultrasound confirmed intact repairs with no neuroma formation. All patients returned to work at a median time of 41.5 days.

**Conclusions:**

Results from this study support the concept of sutureless repair of severed digital nerves using the PGSA polymer-assisted coaptation device by demonstrating that the PGSA polymer-assisted coaptation is safe, effective, and may be considered as an option for nerve approximation.

**Clinical relevance:**

These findings support the potential for broader clinical applications of polymer-assisted sutureless nerve repair.

Injuries to peripheral nerves have a yearly estimated incidence of 13–23 cases per 100,000 persons, and the effect on quality of life and ability to work is a public health concern.^1^, ^2^, ^3^ Following nerve transection, end-end repair using microsurgical suture neurorrhaphy is considered the “gold standard.” Microsurgical repair entails debridement of unhealthy fascicles, accurate tension-free fascicle alignment, and precise epineural suture placement. Suture neurorrhaphy has limitations of trauma to nerve fascicles from the needle, with potential additional scar formation resulting in a physical barrier to axonal regeneration, variable repair strength depending on size and number of sutures used, and variations in tension within the repair causing asymmetric loading and progressive suture failure.^4^, ^5^, ^6^, ^7^ Suture neurorrhaphy is technical and highly dependent on surgeon skills and experience and repair quality may vary widely.^7^^,^^8^

Alternatives to microneurorrhaphy have had a resurgence in recent years.^7^^,^^9^, ^10^, ^11^, ^12^, ^13^, ^14^ Fibrin glue has been successfully used for nerve approximation for several decades; however, dehiscence is possible, and authors have recommended reinforcement of repair with one or two sutures.^15^ As a plasma product, fibrin glue may not be acceptable to some patients because of religious beliefs. Conduits are currently used for nerve repair with small defects but do not completely eliminate use of sutures.^16^ Most recently, a collagen nerve wrap with embedded microscale Nitinol hooks has been used in a cadaver study for approximation of nerve lacerations in the forearm.^10^^,^^11^ There are no clinical results reported with this technique.

A bioresorbable 3-dimensional printed coaptation chamber with light-activated polymer has been reported to secure nerve ends without the use of sutures.^9^ Both the coaptation chamber and the polymer are derived from poly(glycerol sebacate) acrylate (PGSA) (TISSIUM). The nerve repair achieved by the polymer-assisted coaptation device has been shown to be equivalent in tensile strength to microsutures and superior to fibrin glue in a biomechanical study.^9^ This study aimed to evaluate clinical safety and efficacy of sutureless digital nerve repair using this PGSA polymer-assisted coaptation device (COAPTIUM CONNECT®, TISSIUM).

## Materials and Methods

A cohort of adult patients with digital nerve injuries was prospectively enrolled at two centers as part of a first-in-human, single-arm registered clinical trial (NCT04327154) with approval by the human research and ethics committee of the institution. Nerve injuries with a gap or with tendon injuries were excluded. Patients with prior injury to the digit, neuropathy, or medical comorbidity were excluded.

**Figure 1:**
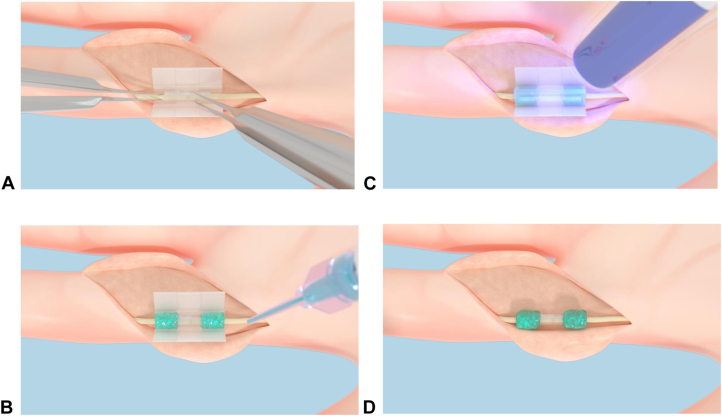
Steps illustrating the application of the sutureless nerve coaptation device. **A** The severed nerve ends are inserted into the coaptation chamber which is positioned in the center of the silicone applicator base. **B** The polymer, in gel form, is applied circumferentially at either end of the nerve-chamber junction. **C** The silicone applicator cap is placed over the construct and the polymer is cured by blue light exposure for 30 seconds. **D** The applicator base and cap are removed, and the repair is complete.

Two experienced fellowship-trained hand surgeons at two centers were trained in the technique and performed the procedure. The injured nerve was exposed using standard incisions; the nerve ends were trimmed when needed and mobilized for 5 mm under operating microscope to allow placement of a silicone background. The nerve ends were placed within a 1.5 mm or 2 mm diameter × 7.5 mm long 3-dimensional printed PGSA coaptation chamber (Fig. 1A). The chamber is a translucent open cylinder with a 1-mm overlap of the ends allowing for molecular diffusion. PGSA polymer was applied circumferentially at either end of the nerve-chamber junction (Fig. 1B) and cured with blue light applied in 30-second cycles for 1 minute to cure the polymer (Fig. 1C) and secure the nerve ends in place (Fig. 1D). Nerve repair integrity was tested by passively moving the finger from full flexion to extension, and routine incision closure was performed. Any gap between the nerve ends within the chamber was visually recorded in millimeters by the surgeon. The “total procedure time” from incision to suture and “nerve repair time,” including mobilization through completion of coaptation, were recorded for each case. After surgery, the patients were allowed controlled active mobilization with a dorsal splint to avoid hyperextension for a period of 3 weeks.

The patients were prospectively followed for 12 months and seen at intervals of 2 and 6 weeks and 3, 6, and 12 months. Pain (visual analog scale (VAS) score), sensory recovery (static 2-point discrimination (2PD), and Semmes-Weinstein monofilament testing (SMWF)), and Impact of Hand Nerve Disorders (I-HaND v2.0) score were recorded. High-resolution ultrasound imaging was used to confirm the integrity of the repair within the coaptation chamber. Subjects were monitored for any adverse events or complications.

### Cumulative incidence of complications

Safety of the PGSA polymer-assisted coaptation device was assessed by collecting and evaluating the cumulative incidence of complications related to the investigational device. This included infection, pain lasting >3 months, excessive inflammation as determined by the investigator, device extrusion, impaired wound healing, allergic reaction, serious adverse device effects, and symptomatic neuroma formation.

### Functional assessment of nerve repair

Efficacy of nerve repair was assessed using SWMF and static 2PD at regular intervals of 6 weeks, 3 months, 6 months, and 12 months postsurgery. SWMF testing was performed using a standardized set of filaments applied to the injured digit.^17^, ^18^, ^19^ 2PD results were converted to Modified Highet classification.^20^

### Patient-reported outcomes

Patient-reported outcomes were assessed by VAS score for pain and the I-HaND (v2.0) scale. The VAS score for pain was marked on a 0−10 line by patients, with higher scores indicating a higher level of pain. The value recorded was multiplied by 10 to present a value from 0 to 100, and this value was used for analysis purposes.

The I-HaND scale (v2.0) is a patient-reported measure of the impact of nerve trauma in the hand and provides an overall quantitative assessment of symptoms and functional difficulties.^21^ The I-HaND (v2.0) consists of 32 questions, each measured on a scale of 1−5, with a score of 1 representing the “best” and 5 representing the “worst” outcome. An overall score was derived by adding the values of all valid responses and dividing by the number of valid responses, which resulted in an average score between 1 and 5, and then this score was converted to a percentage. Lower scores indicate better patient-reported outcomes. Additionally, return to work was periodically assessed as part of the study follow-up.

### Imaging of nerve coaptation

High-resolution ultrasound scanning of the nerve repair was performed by an independent musculoskeletal radiologist at 3, 6, and 12-month postinjury in both longitudinal and transverse planes to assess continuity of the nerve repair construct.

### Surgeon use questionnaire

After each procedure, surgeon use questionnaires were administered to the operating surgeon to gain insight into the user experience.

### Analysis methods

All analyses and analysis methods were prespecified as part of the study protocol. Because of the population size, all analyses are descriptive. Continuous variables are presented as means and standard deviations. Categorical variables are presented as numbers and percentages. All analyses were conducted using SAS, version 9.4 or higher (SAS Institute, Inc).

## Results

**Figure 2:**
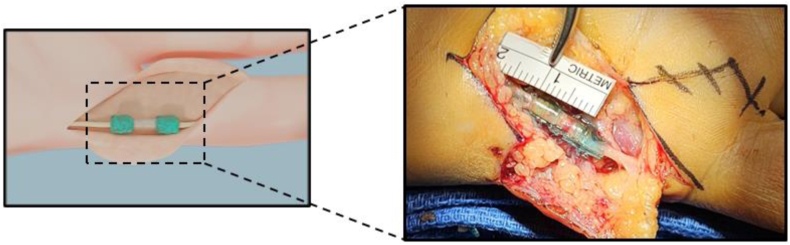
Intraoperative photograph of repaired ulnar digital nerve of small finger showing nerve ends approximated within the clear polymer chamber and secured by cuff of cured polymer gel at the nerve-chamber junction on either side.

A total of 14 patients were consented to the study. One patient was breast feeding and failed preoperative screening, and one patient had an intact nerve at exploration and was an intraoperative screen failure. The remaining 12 patients underwent nerve repair with the technique described above. Procedural success, defined as successful nerve coaptation at surgery was achieved in all cases (n = 12, 100%). An example image of the repair is shown in the Figure 2. Ten patients completed 12 months’ follow-up. Two patients were lost to follow-up: one after the week 2 follow-up visit and the other after the week 6 follow-up visit. The patient and injury details are listed in the Table. All injuries were from sharp lacerations located in flexor zone 1 or 2. The mean total procedure time, inclusive of gaining surgical access, irrigation, debridement, wound closure, and any other repairs, was 63.3 (± 16.3) minutes with a mean nerve repair time (time from start of nerve mobilization to a completed and tested repair) of 26.6 (± 12.0) minutes. The median nerve gap visualized through the translucent chamber after repair completion as measured by the surgeon with a sterile ruler was 1 mm (Fig. 2). All 10 patients recovered full flexion and extension of the injured digit at final follow-up.

**Table 1:** Patient Characteristics, Injury, and Procedure Information

Patient Number Total = 12	Age (y)	Sex	Injury Location	Injury Flexor Zone	Injury Etiology and Mechanism	Time From Injury to Repair (days)	Gap After Repair (mm)
001	27.6	Female	Left index finger	1	Domestic accident	3	1
			Radial digital nerve		Knife		
002	32.8	Male	Left middle finger	1	Domestic accident	8	0
			Radial digital nerve		Knife		
004	20.4	Male	Right index finger	1	Domestic accident	5	0
			Ulnar digital nerve		Knife		
005	18.1	Male	Right index finger	2	Work accident	11	2
			Radial digital nerve		Knife		
006	26.2	Male	Right index finger	2	Domestic accident	4	1
			Ulnar digital nerve		Glass		
007	29.4	Male	Left index finger	2	Sport injury	2	0
			Radial digital nerve		Knife		
008	22.9	Male	Right middle finger	2	Domestic accident	1	1
			Radial digital nerve		Porcelain plate		
010	19.1	Female	Left middle finger	1	Work accident	6	1
			Radial digital nerve		Broken glass		
011	19.3	Male	Left thumb	2	Domestic accident	5	1
			Radial digital nerve		Metal sheet		
012	49.2	Male	Left small finger	2	Work accident	10	0.5
			Ulnar digital nerve		Metal door		
013	45.2	Male	Left middle finger	1	Work accident	7	1
			Radial digital nerve		Angle grinder		
014	51.2	Female	Left small finger	2	Domestic accident	10	0
			Ulnar digital nerve		Ceramic doorknob		

### Cumulative incidence of complications

No prespecified events or adverse events were reported throughout the follow-up duration.

### Functional assessment of nerve repair

**Figure 3:**
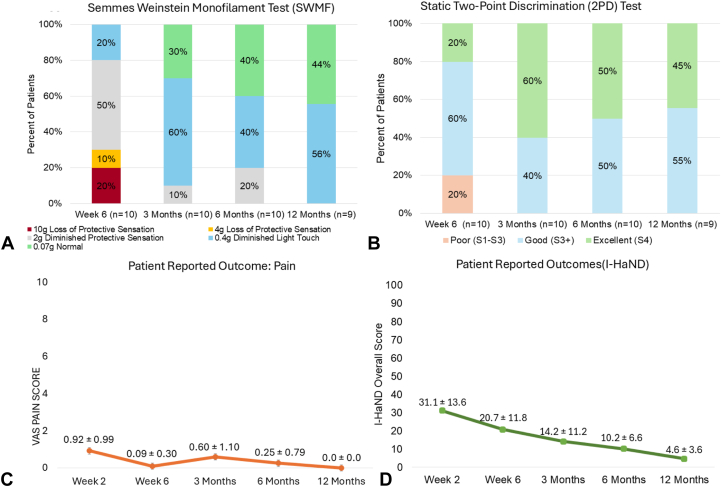
Patient outcomes **A** Semmes-Weinstein Monofilament, displayed as a percentage of patients in each filament category, shows an improvement in functional nerve recovery from 6 weeks to 3 months, which is maintained to 12 months. **B** Static 2-point discrimination (2PD) test demonstrates that all patients have a good or excellent meaningful recovery by 3 months, which is maintained throughout the study follow-up duration. **C** The mean visual analog scale (VAS) pain score (orange, on 0−10 cm scale) decreased from 0.92 at week 2 to 0.00 at 12 months. **D** Impact of hand nerve disorders (I-HaND) as measured by the I-HaND v2.0 Questionnaire (green) shows a reduction over the study follow-up duration.

All 10 patients reported recovery of sensation at the final follow-up. One patient attended their 12-month follow-up visit; however, 2PD and SWMF assessments were not recorded by the hand therapist. SWMF at 12 months in nine patients achieved a value of 0.4g (“diminished light touch”) or better (Fig. 3A). The patient who did not have a 12-month measurement had a score of 0.4g (“diminished light touch”) at 6 months. The mean SWMF score improved from 3.48g ± 3.58g at 6 weeks to 0.59g ± 0.76g at 6 months and 0.25g ± 0.17g at 12 months. The Figure 3B depicts the percentage of patients with each static 2PD modified Highet score at the four measurement points and showed progressive improvement. All nine patients assessed at 12 months had a score of “Good” (S3+) or “Excellent” (S4).

### Patient-reported outcomes

Both the VAS for pain and I-HaND questionnaire showed improvement over study follow-up period (Fig. 3C). The mean VAS pain score was 0 (zero) at 12 months with no patient reporting pain. Improvement in patient-reported outcomes, as measured by the I-HaND scale (v2.0), was observed with the mean I-HaND overall score decreasing from 31.1% ± 13.6% at the 2-week visit to 4.6% ± 3.6% at the 12-month visit (Fig. 3D). All patients returned to their previous occupation by 3 months from nerve repair, with four returning as soon as 2 weeks. The median time to return to work was 41.5 days.

### Imaging of nerve coaptation

**Figure 4:**
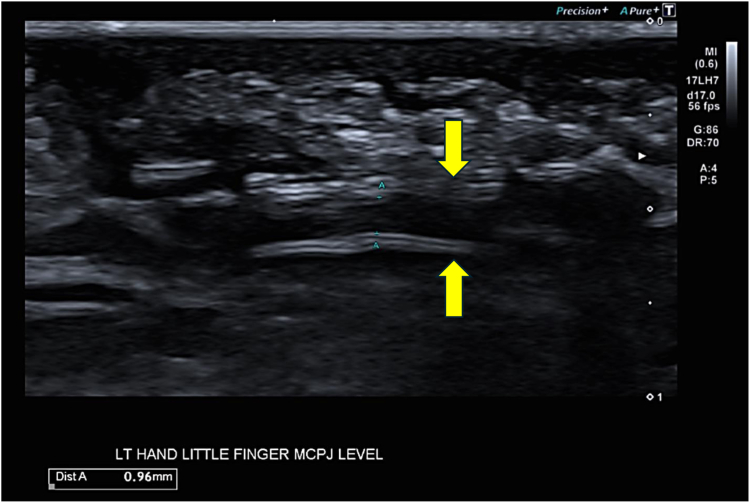
Ultrasound image of the coaptation site at 1 year. Coaptation at the level of the MP joint. Arrows denote the nerve-chamber, and the nerve can be seen in continuity within the chamber.

Ultrasound images reported intact repair with the proximal and distal nerve ends both located within the visualized coaptation chamber (Fig. 4). At the final 1-year follow-up visit, the radiologist could confirm nerve continuity in all cases.

### Surgeon use questionnaire

The overall perception of the surgeons using the sutureless coaptation system, when compared to suture neurorrhaphy, was considered easier in five cases (42%), similar in five cases (42%), and harder in two cases (17%) because of limited access in distal lacerations. In all 12 cases (100%), surgeons were satisfied with the extent of nerve end manipulation required to perform the repair, including seven cases where surgeons believed the repair required less nerve handling compared to suture repair (58.3%). Subjectively, the surgeons reported that the fixation strength of the coaptation was considered either the same (41.7%) or superior to microsuture (58.3%).

## Discussion

The nerve coaptation device evaluated in this cohort was designed to eliminate the need for sutures in nerve repair. With neurorrhaphy, nerve ends must be properly aligned and approximated to achieve satisfactory regeneration with minimal sutures. The trauma imposed via the needle and sutures can create inflammation, and the sutures within the repaired nerve structure can create an area of stress concentration with additional fibrous tissue reaction.^22^ Additionally, the tension of the suture across the repair site can distort fascicle alignment^7^ and inhibit axon regeneration.^23^ Suture techniques also have limitations, for example, variations in suture tension within a repair may cause asymmetric loading and progressive suture failure. Suturing is a technical process and highly dependent on the surgeon, therefore, leading to variable results from one nerve repair to another.^7^^,^^8^

In this clinical study, sutureless coaptation was achieved in all cases. Subjectively, the surgeons reported the technique was as easy or easier than microneurorrhaphy in most cases. When compared to landmark studies on digital nerve repair, polymer-assisted repairs performed in this study fared well.^24^, ^25^, ^26^, ^27^ Nerve recovery as measured by the Al-Ghazal 10-point scale in previous studies showed Excellent recovery in 12.5% to 17% of patients, Good recovery in 39.1% to 51.1% of patients, Fair recovery in 22.8% to 31.2% of patients, and Poor recovery in 9.1% to 17.2% of patients.^24^^,^^25^ Similarly, final outcomes observed via static 2PD assessments ranged from 13% to 34% for Excellent recovery, 54% to 61% for Good recovery, and 12% to 26% for Poor recovery.^24^, ^25^, ^26^, ^27^ When compared with the outcomes of polymer-assisted repairs in this study, where all patients had Good (55%) or Excellent (45%) final outcomes as assessed by static 2PD, this atraumatic method of repair seems to be a promising alternative to traditional microsuture repair.

The PGSA polymer-assisted coaptation device includes potential benefits associated with conduit repair such as contained repair site with less ingress of scar, better axonal alignment, and no axonal escape.^13^^,^^14^^,^^28^ Further studies are needed to confirm these benefits. The quality of repair using this technique may have less variation with surgeon training and experience. Additionally, if the completed repair is unsatisfactory, it can be revised by gently removing the cured polymer piecemeal without disturbing the epineurium.

This study is not without limitations. The sample size is small, but appropriate for a first-in-human study.^29^ While the outcome of digital nerve repair may not directly translate to larger mixed nerves, the outcomes and absence of adverse reaction to the material in a subcutaneous location recovery demonstrate the technique is safe and does not impair nerve regeneration. Although a new technique, it is not entirely unfamiliar to surgeons as the application of fibrin glue and nerve conduit wrap are routinely employed techniques.

Results from this pilot study provide clinical evidence that coaptation using the PGSA polymer-assisted device of severed digital nerves amenable to primary repair is safe and patients experienced a high level of functional recovery assessed via SWMF and static 2PD tests by 6 months that was maintained to 12 months. There were no complications related to the study device reported throughout the study duration, and repair procedures were successful in all patients available for follow-up. There was no incidence of neuroma formation and no reoperations, and study patients experienced low levels of pain postrepair, with no patients reporting pain by the 12-month follow-up visit. This study has shown it is possible to do a sutureless nerve repair in a digital nerve model that may be translatable to additional nerve types.

## Conflicts of Interest

Dr Brett is an employee of the sponsor of the clinical study. Dr Power has a consulting agreement with TISSIUM for surgeon usability testing and medical monitor role for study. Dr Bindra, has a consulting agreement with TISSIUM for surgeon and sales training. No benefits in any form have been received or will be received by the other authors related directly to this article.
